# Inhibition of HOXD11 promotes cartilage degradation and induces osteoarthritis development

**DOI:** 10.1186/s13018-024-04573-7

**Published:** 2024-02-02

**Authors:** Quan Hong, Zhong-Xun Liu, Hai-Feng Liang, De-Guang Wu, Yan Chen, Bo Yu

**Affiliations:** 1 Department of Orthopedics, Zhujiang Hospital, Southern Medical University, Guangzhou, 510282 Guangdong China; 2 Department of Ultrasonic Diagnosis, Zhujiang Hospital, Southern Medical University, Guangzhou, 510282 China; 3 Department of Orthopedics, Jieyang People’s Hospital (Jieyang Affiliated Hospital, Sun Yat-Sen University), Jieyang, 522000 Guangdong China

**Keywords:** *5′-HOXD* genes, *HOXD11*, Cartilage degradation, Osteoarthritis

## Abstract

The *5′-HOXD* genes are important for chondrogenesis in vertebrates, but their roles in osteoarthritis (OA) are still ambiguous. In our study, *5′-HOXD* genes involvement contributing to cartilage degradation and OA was investigated. In bioinformatics analysis of *5′-HOXD* genes, we obtained the GSE169077 data set related to OA in the GEO and analyzed DEGs using the GEO2R tool attached to the GEO. Then, we screened the mRNA levels of *5′-HOXD* genes by quantitative reverse transcriptase–polymerase chain reaction (qRT-PCR). We discovered that OA chondrocyte proliferation was inhibited, and apoptosis was increased. Moreover, it was discovered that SOX9 and COL2A1 were downregulated at mRNA and protein levels, while matrix metalloproteinases (MMPs) and a disintegrin-like and metalloproteinase with thrombospondin motifs (ADAMTSs) were upregulated. According to the results of differentially expressed genes (DEGs) and qRT-PCR, we evaluated the protein level of *HOXD11* and found that the expression of *HOXD11* was downregulated, reversed to MMPs and ADAMTSs but consistent with the cartilage-specific factors, SOX9 and COL2A1. In the lentivirus transfection experiments, *HOXD11* overexpression reversed the effects in OA chondrocytes. In human OA articular cartilage, aberrant subchondral bone was formed in hematoxylin–eosin (H&E) and Safranin O and fast green (SOFG) staining results. Furthermore, according to immunohistochemistry findings, SOX9 and *HOXD11* expression was inhibited. The results of this study established that *HOXD11* was downregulated in OA cartilage and that overexpression of *HOXD11* could prevent cartilage degradation in OA.

## Introduction

Osteoarthritis (OA) is a prevalent degenerative joint illness which can cause disability and severely harm human health, affecting approximately 3.6% of the global population [1]. Cartilage degeneration, characterized by gradual loss of articular cartilage, reconfiguration of subchondral bones, as well as osteophyte formation at joint borders, is main pathological hallmark of OA joints [2, 3]. However, the exact mechanism of occurrence and development of OA has not yet been fully elucidated. OA has become widely accepted as a complex degenerative disease affecting the entire joint rather than merely a mechanical cartilage damage condition [3, 4].

There are two components of articular cartilage: chondrocytes and extracellular matrix (ECM) [5]. Among transcription factors, SOX9, with other transcription factors such as runt-related transcription factor 2 (RUNX-2), hypoxia and hypoxia-inducible factor (HIF-1), as well as forkhead box O3A (Foxo3A), is involved in cartilage phenotype regulation and has an essential function in cartilage ECM homeostasis and chondrocyte survival [6–10]. In chondrocytes, if balance between catabolism and anabolism of SOX9 is disrupted, it increases matrix metalloproteinase-3 (MMP3) expression and decreases COL2A1 expression, leading to the destruction of cartilage ECM homeostasis; therefore, the SOX9 stability is crucial for articular cartilage [5]. In addition to MMPs, another matrix-degrading enzyme of a disintegrin and metalloproteinase with thrombospondin motifs (ADAMTSs) is highly expressed in OA joint, suggesting their involvement in matrix degradation during OA development [11, 12].

In vertebrates, homeobox genes (*HOX* genes) have an essential involvement in cell differentiation regulation, body patterning, as well as cell migration by regulating target gene expression [13]. The *HOXD* gene family’s significance in vertebrate limb development has been widely researched. In vertebrate limb development, *5′-HOXD* gene (*HOXD9-13*) is required for establishing patterning and is expressed in a nested manner along the A–P axis, it can be divided into three sections patterned along the proximal–distal (P–D) axis: the stylopod (humerus and femur), the zeugopod (radius/ulna and tibia/fibula), and the autopod (the wrist/forepaw, ankle/hindpaw) [14, 15]. *HOXD9* and *HOXD10* function in the stylopod region [16, 17], *HOXD11* in the zeugopod region [16], and *HOXD12* and *HOXD13* in the autopod region [18]. An abnormally expressed gene can result in dramatic, region-specific limb deformities. For example, a polyalanine expansion in the N-terminus of the *HOXD13* gene results in polydactyly in humans [19], and idiopathic congenital clubfoot is associated with mutations in the *HOXD12* and *HOXD13* [20]. Since many *5′-HOXD* genes have extensive functional overlap, it is difficult to completely detect the role of a single member [21]. One of the *5′-HOXD* genes, *HOXD9*, is elevated in synovial cells of rheumatoid arthritis patients. *HOXD9* may play a significant involvement in retinoic acid (RA) because of its association with synovial cell proliferation [22, 23]. *HOXD9* has been implicated in chondrogenesis through regulating SOX9 and COL2A1 directly, according to our previous studies [24]. However, no direct genetic evidence links *5′-HOXD* genes to OA development.

As mentioned above, we hypothesized that one or more *5′-HOXD* genes may be associated with cell proliferation, cell apoptosis, and expression of cartilage-specific factors involved in cartilage degradation and inducing osteoarthritis development. In this study, we aimed to investigate if *5′-HOXD* genes were involved in cartilage degradation and OA development. First, we collected and separated cartilage samples from normal and OA patients and cultured the primary chondrocytes. We then examined cell proliferation by CCK-8 analysis, cell apoptosis by flow cytometric analysis, and cartilage-specific factors (SOX9-COL2A1) expression by quantitative reverse transcriptase-polymerase chain reaction (qRT-PCR) and Western blot (WB) in normal and OA chondrocytes. We identified *5′-HOXD* genes (*HOXD9-13*) that were differentially expressed (DE) in OA chondrocytes through bioinformatic analysis using Gene Expression Omnibus (GEO) database. Next, we investigated differentially expressed genes (DEGs) expression by qRT-PCR and WB analysis. Finally, we explored whether overexpression of *HOXD11* using lentivirus could prevent cartilage degradation in OA.

## Materials and methods

### Human articular cartilage samples and chondrocyte culture

Normal human cartilage specimens were extracted and separated from patients’ knee joints (*n* = 6) who had suffered above-knee amputations after severe lower extremity injuries. Human cartilage samples were taken from knee joints of OA patients (*n* = 6) who had underwent total knee arthroplasty (TKA). Southern Medical University’s Institute Research Ethics Committee reviewed and approved this study. Patients/participants gave their explicit written consent to take part in our research.

Primary chondrocytes were separated from the cartilage of the human tibial plateau and rinsed with Dulbecco’s modified Eagle’s media (DMEM; Sigma, St., USA) according protocol as previously described. Furthermore, cartilage tissue specimens were isolated and divided into fragments, before being treated with 0.25% trypsin for 30 min and 0.2% collagenase type II for 8 h at 37 °C (Sigma-Aldrich, USA). The digest was diluted in DMEM with 10% (v/v) fetal bovine serum (FBS) (Hyclone, USA), 1% penicillin/streptomycin (v/v) (Invitrogen, USA), 2 mM glutamine (Sigma-Aldrich, USA), as well as 50 g/mL ascorbic acid (Sigma-Aldrich, St. USA). Cells were plated in a Petri dish at a density of 1 × 10^5^ cells/mL as well as cultivated at 37 °C in an incubator of 95% O_2_ and 5% CO_2_. Cultivated chondrocytes were used in subsequent experiments when cells were at 80% confluence.

### Analysis of cell proliferation

A cell counting kit-8 assay (CCK-8; Beyotime, China) was utilized to evaluate cell proliferation based on manufacturer’s instruction described in the previous study. Cells were plated in 6-well plates at a density of 1 × 10^5^ cells/well as well as incubated for confluence, then measured at 0, 1, 3, 5, and 7 d. A microplate reader (Bio-Rad, USA) was utilized to measure optical density (OD) at 450 nm.

### Flow cytometric analysis

An Annexin V-fluorescein isothiocyanate (FITC) apoptosis detection kit (BD Biosciences, USA) was utilized to evaluate percentage of apoptotic cells following manufacturer’s instructions. Briefly, chondrocytes were seeded in 6-well plates at a density of 1 × 10^5^ cells/well as well as incubated until confluence, after which cells were washed with 4 °C phosphate buffer saline (PBS) thrice and fixed at −20 °C for 1 h at least in ethanol. After thrice washes, cells were incubated in dark for 30 min after being treated with 10 μL of Annexin V-FITC and 5 μL of propidium iodide (PI). Then, 0.6 mg/mL RNase in PBS with 0.5% (v/v) Tween 20 as well as 2% FBS was added to cells. A FACSCalibur flow cytometer (BD Bioscience, USA) was used to detect stained cells using CellQuest software. Approximately 1 × 10^4^ cells of each sample were counted. Utilizing WinMDI software, data were analyzed (version 2.9, Bio-Soft Net).

### qRT-PCR

Total RNA was separated utilizing a TRIzol reagent (Invitrogen, USA). Utilizing a PrimeScript RT Reagent Kit (Takara Bio, China), first-strand cDNA synthesis was accomplished. qRT-PCR was conducted utilizing SYBR Premix Ex Taq (Takara Bio, Beijing, China) based on manufacturer’s instructions on a LightCycler 480 SYBR Green I Master (Roche, Indianapolis, USA) at 95 °C for 10 min, 40 cycles at 95 °C for 15 s and 60 °C for 1 min. Performed genes and primer sequences are registered in Table 1. Gene expression was normalized to glyceraldehyde-3-phosphate dehydrogenase (GAPDH).

**Table 1:** qRT-PCR primer sequence and cycling conditions

Gene	NCBI Gene ID	GenBank Accession	Primer Sequence (Forward/Reverse)5′ → 3′	TM (℃)	Amplicon Size (bp)
SOX9	6662	NM_000346	AGCGAACGCACATCAAGAC	60.7	85
			CTGTAGGCGATCTGTTGGGG	62	
COL2A1	1280	NM_001844	TGGACGATCAGGCGAAACC	62	244
			GCTGCGGATGCTCTCAATCT	62.4	
HOXD9	3235	NM_014213	GGACTCGCTTATAGGCCATGA	61.1	141
			GCAAAACTACACGAGGCGAA	60.9	
HOXD10	3236	NM_002148	GACATGGGGACCTATGGAATGC	62.5	129
			CGGATCTGTCCAACTGTCTACT	60.9	
HOXD11	3237	NM_021192	TCGACCAGTTCTACGAGGCA	62.5	325
			AAAAACTCGCGTTCCAGTTCG	61.7	
HOXD12	3238	NM_021193	CAGTCGCCAGACTCTTTCTACT	61.1	235
			GCTCTTCGGGTCCGTCTTT	61.7	
HOXD13	3239	NM_000523	CTTCGGCAACGGCTACTACAG	62.8	124
			TGACACGTCCATGTACTTCTCC	61.4	
MMP3	4314	NM_002422	AGTCTTCCAATCCTACTGTTGCT	61	226
			TCCCCGTCACCTCCAATCC	63	
MMP13	4322	NM_002427	ACTGAGAGGCTCCGAGAAATG	61.3	103
			GAACCCCGCATCTTGGCTT	62.7	
ADAMTS4	9507	NM_005099	GAGGAGGAGATCGTGTTTCCA	60.9	118
			CCAGCTCTAGTAGCAGCGTC	61.8	
ADAMTS5	11,096	NM_007038	GAACATCGACCAACTCTACTCCG	62.3	107
			CAATGCCCACCGAACCATCT	62.5	
GAPDH	2597	NM_001256799	GGAGCGAGATCCCTCCAAAAT	61.6	197
			GGCTGTTGTCATACTTCTCATGG	60.9	

### Western blot

Cells were rinsed with 4 °C PBS thrice as well as added with radioimmunoprecipitation assay (RIPA) buffer (Beyotime, China) with 1% phenylmethylsulfonyl fluoride (Sigma, St. USA). Moreover, 10% SDS–polyacrylamide gel electrophoresis (SDS-PAGE) was utilized to extract total protein, and then, proteins were translocated to polyvinylidene difluoride membranes (Thermo-Fisher, Hampton, NH). Primary antibodies against ADAMTS4 (1:500 dilution, Proteintech, 11865-1-AP), ADAMTS5 (1:1000 dilution, Abcam, ab41037)), MMP3 (1:500 dilution, Proteintech, 17873-1-AP), MMP13 (1:500 dilution, Proteintech, 18165-1-AP), SOX9 (1:1000 dilution, Cell Signaling Technology, 82630), COL2A1 (1:1000 dilution, Abcam, ab34712), HOXD11 (1:500 dilution, Proteintech, 18734-1-AP), and GAPDH (1:1000 dilution, Proteintech, 60004-1-Ig) were diluted at appropriate concentration and incubated with membranes at 4 °C overnight. Horseradish peroxidase (HRP)-conjugated anti-rabbit immunoglobulin G (Santa Cruz Biotechnology, sc-2004) or anti-mouse IgG (Santa Cruz Biotechnology, sc-2005) was diluted at 1:3000 and incubated with the membranes at RT for one hour. LI-COR Imaging System (Biosciences, USA) was utilized to visualize protein bands. The band intensities were assessed utilizing ImageJ analysis (National Institutes of Health, USA). Each gene’s band intensity was normalized to GAPDH.

### Cell transfection

GenePharma (Shanghai, China) supplied HOXD11 overexpressing lentivirus (OE-HOXD11) and control vector. Lentivirus was transfected into human chondrocytes based on manufacturer’s instruction. Cells were transfected for 48 h, and then, following experiments were performed.

### Immunohistochemistry and histomorphometry analysis

The OA and normal human articular cartilage were treated with 10% buffered formalin for fixation and decalcified with 10% (w/v) EDTA; pH 7.4 for three weeks and then embedded with paraffin. Sections were cut into 6 μm thickness as well as further dyed with anti-SOX9 (1:500 dilution, Cell Signaling Technology, 82630), anti-HOXD11 (1:300 dilution, Proteintech, 18734-1-AP) at 4 °C overnight, then stained with second antibodies conjugated with HRP (Santa Cruz Biotechnology, CA, USA). Microscope BX51 (Olympus, Japan) was utilized to obtain images. ImageJ analysis software was utilized to quantify the intensity of *HOXD11* and SOX9 expression (National Institutes of Health, USA). For histomorphometry, six-micrometer-thick sections of knee cartilage were dyed with H&E, Safranin O and fast green (SOFG) to evaluate cartilage damage. ImageJ was used to quantify safranin O loss in relation to total cartilage. Histological assessment was evaluated based on Osteoarthritis Research Society International (OARSI) histological scoring system and performed by two blinded observers.

### Analysis of DEGs

We searched for data sets related to OA in the GEO and obtained the GSE169077 data set, which contained knee cartilage samples from five normal humans as well as six OA patients. Then, we analyzed DEGs using the GEO2R tool attached to the GEO. As the criteria of |log_2_(FC)|> 1 and *P* < 0.05, DEGs were screened from the GSE169077 data set.

### Statistical analysis

Data were presented as the means ± standard deviations. All the statistical analyses were performed utilizing SPSS 13.0 (SPSS, Chicago, USA). Unpaired Student’s *t* test was utilized for analyzing the differences between two groups. We used one-way analysis for variance and Tukey’s test for multiple comparisons. Probability (*P*) values > 0.05 were considered statistically significant.

## Results

### Inhibited proliferation and enhanced apoptosis in human OA chondrocytes

**Fig. 1:**
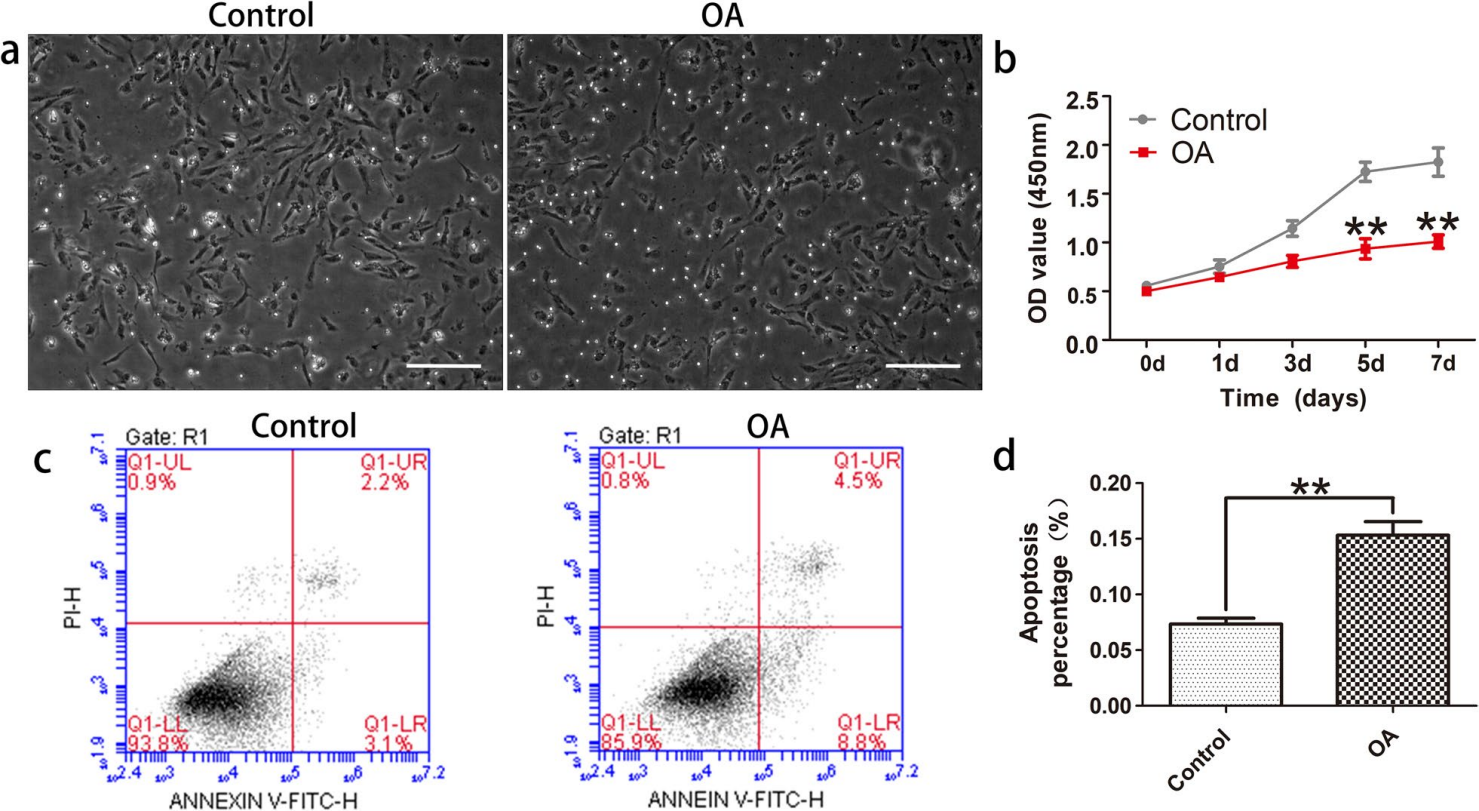
Cell proliferation and apoptosis in OA and normal chondrocytes. **a** OA and normal chondrocyte cells were cultivated in 60 mm plates (magnification, × 100). Scale bars: 200 μM. **b** Cell absorbances (450 nm) were estimated at 0, 1, 3, 5, and 7 d by CCK8 assay. **c** Flow cytometry with Annexin V-FITC/PI dual dying was utilized to quantify OA cells and normal chondrocytes apoptosis. **d** Apoptosis percentage between OA cells and normal chondrocytes. Data are presented as mean ± SD of at least three independent experiments. *P < 0.05 and **P < 0.01 as relative with control

We isolated primary chondrocytes from OA and normal knee articular cartilage, then compared cell absorbance at 450 nm, to evaluate proliferation effects in OA chondrocytes (Fig. 1a, b). The results depicted inhibited proliferation in OA chondrocytes as relative to control group, a 45.75% reduction at 5 d and a 44.69% reduction at 7 d, they were both reduced significantly (*P* < 0.05). We further investigated the effects of cell apoptosis in OA chondrocytes using flow cytometry analysis (Fig. 1c, d). Results revealed that the apoptotic percentage was 15.32% in OA and 7.35% in the controls (*P* < 0.05). These results suggested that cell proliferation was suppressed, and apoptosis was improved in OA chondrocytes.

### Reduced expression of specific factors in human OA chondrocytes

**Fig. 2:**
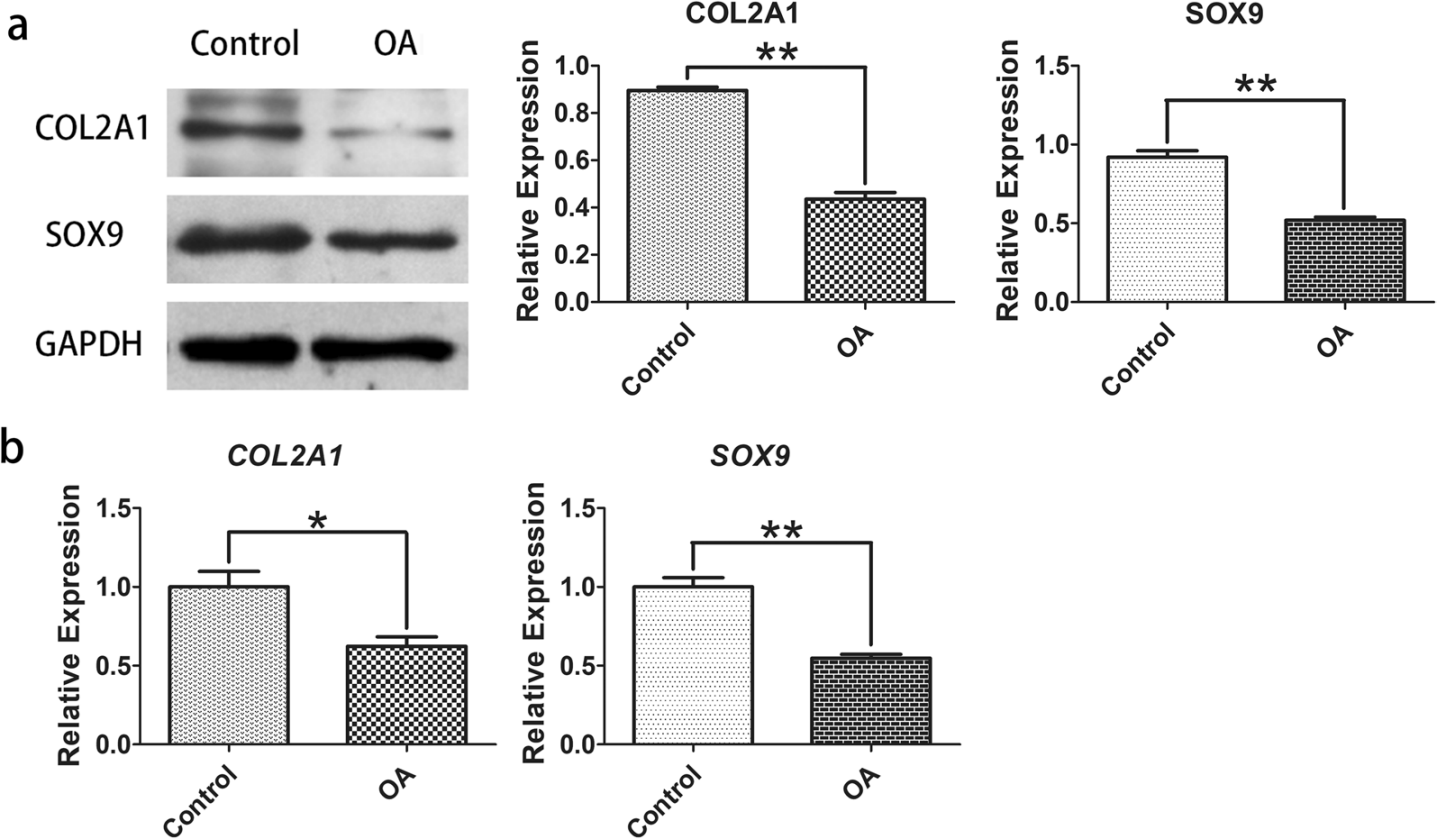
SOX9 and COL2A1 expression in OA cells and normal chondrocytes. **a** WB was carried out to quantify SOX9 and COL2A1 protein levels in OA cells and normal chondrocytes. **b** qRT-PCR was performed to quantify SOX9 and COL2A1 mRNA level. *P < 0.05 and **P < 0.01 as relative with control

Cartilage-specific markers: SOX9 and COL2A1, have been identified as dominant transcription factors necessary for chondrogenesis [25]. In qRT-PCR assays, SOX9 and COL2A1 mRNA levels in OA were reduced than controls (*P* < 0.05 vs controls) (Fig. 2b). WB assays displayed that SOX9 and COL2A1 were reduced in OA compared to controls (*P* < 0.05) (Fig. 2a). These findings indicated that SOX9 and COL2A1 expressions were inhibited in OA chondrocytes.

**Fig. 3:**
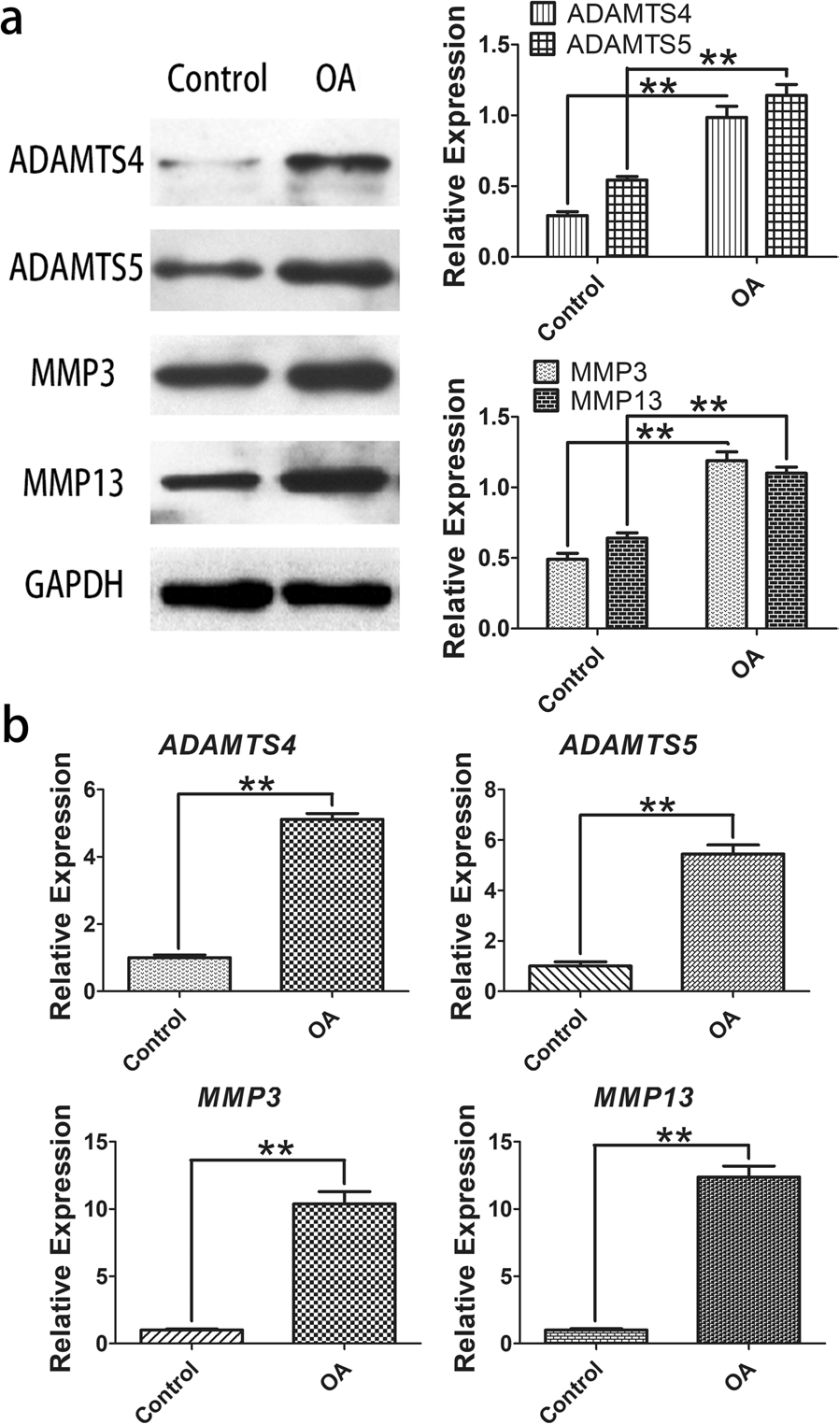
Expression of ADAMTSs (ADAMTS4 and ADAMTS5) and MMPs (MMP3 and MMP13) in OA cells and normal chondrocytes. **a** WB was carried out to evaluate ADAMTSs and MMPs in OA cells and normal chondrocytes. **b** qRT-PCR was performed to quantify mRNA level of ADAMTSs and MMPs. *P < 0.05 and **P < 0.01 as relative with control

For the expressions of ADAMTSs and MMPs, qRT-PCR and WB were utilized to study mRNA and protein levels. The findings exposed that mRNA levels of ADAMTSs (ADAMTS4, ADAMTS5) and MMPs (MMP3, MMP13) were upregulated in OA than in control (Fig. 3b). Moreover, WB assays exposed upregulated protein levels of ADAMTSs (ADAMTS4, ADAMTS5) and MMPs (MMP3, MMP13) in OA compared with control (*P* < 0.05) (Fig. 3a).

### Downregulated *HOXD11* in human OA chondrocytes

**Fig. 4:**
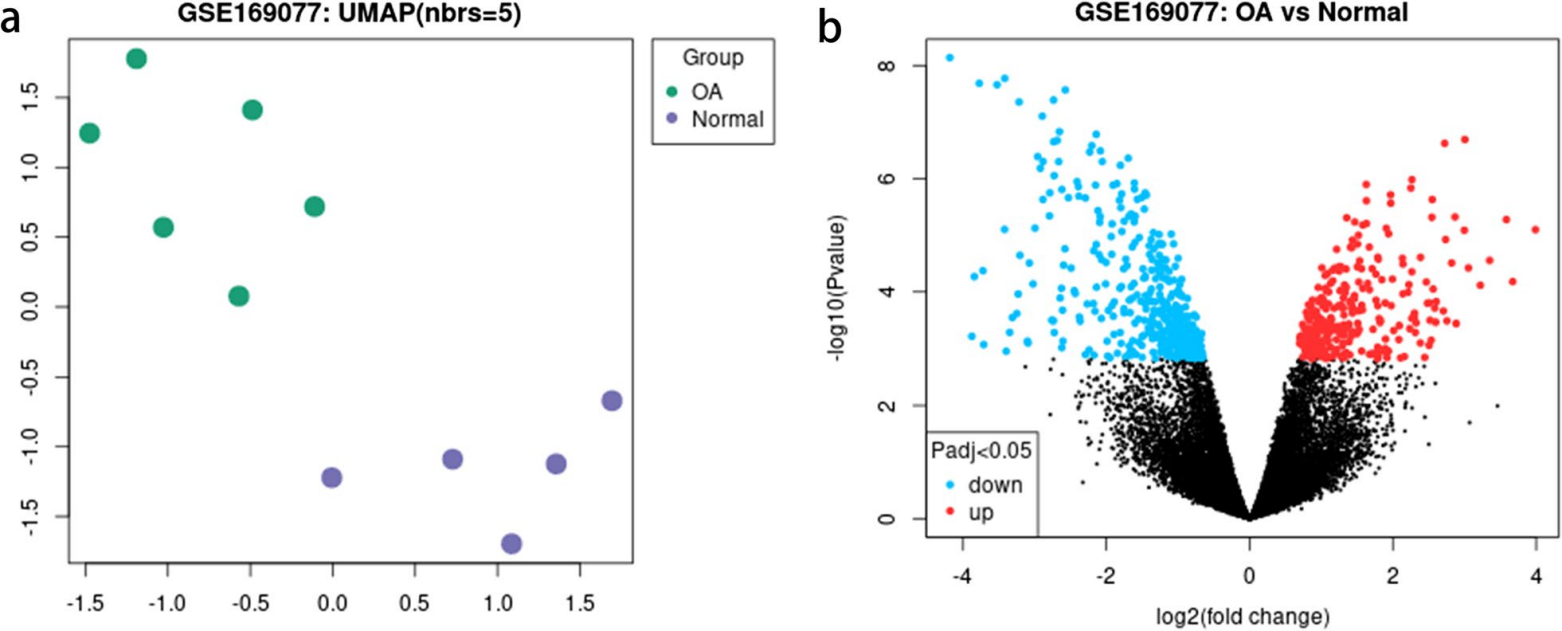
Identification of DEGs analysis. **a** UMAP plot of knee cartilage samples in GSE169077 data set. The purple dot represents knee cartilage samples of five normal humans, and the green dot represents knee cartilage samples of six OA patients. **b** Volcano plot of DEGs in GSE169077 data set. The red dot indicates elevated genes (*n* = 720), whereas blue dot indicates downregulated genes (*n* = 810)

Given the importance of *5′-HOXD* genes in chondrogenesis, we analyzed the DEGs in *HOXD* genes using the GEO data set. As shown in the Uniform Manifold Approximation and Projection (UMAP) diagram (Fig. 4a), the differences in gene expression levels between the knee cartilage samples of normal people and the OA patients can be distinguished well. Through the analysis of GEO2R, we obtained 720 genes upregulated and 810 genes downregulated in OA patients, shown in the volcano diagram (Fig. 4b). Given master involvement of *HOXD* genes in vertebrate limb development, we concentrated *HOXD* genes expression. In *HOXD* genes, the chip included *HOXD4*, *HOXD9*, *HOXD11*, and *HOXD13* (Table 2). Among them, *HOXD11* met the screening criteria for DEGs, the *P* value was 0.0161, and ǀlog_2_FCǀ was 1.31709. Therefore, *HOXD11* was selected as an important DEG in our further analysis.

**Table 2:** The DEGs

Gene.ID	Gene.symbol	*P* value	ǀLog_2_FCǀ	Up/down
205605_at	HOXD9	0.203	0.584441	Down
207397_s_at	HOXD13	0.443	0.547257	Down
206601_s_at	LOC401021///HOXD4///HOXD3	0.451	−0.35956	Down
214604_at	HOXD11	0.0161	−1.31709	Down

**Fig. 5:**
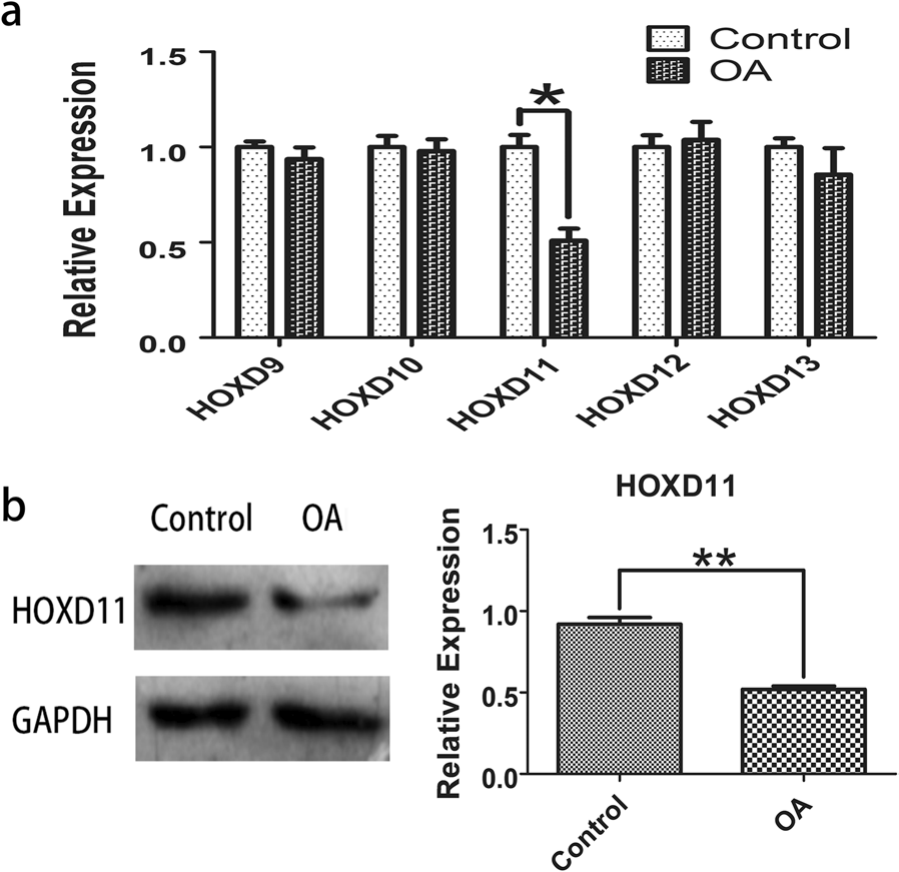
Expression of *HOXD11* in OA cells and normal chondrocytes. **a** qRT-PCR was conducted to quantify *5′-HOXD* mRNA levels (*HOXD9*, *HOXD10*, *HOXD11*, *HOXD12*, and *HOXD13)*. **b** WB was conducted to quantify *HOXD11* protein levels in OA cells and normal chondrocytes. *P < 0.05 and **P < 0.01 as relative with control

To verify the DEGs, we examined the effects of *5′-HOXD* genes in OA chondrocytes, and qRT-PCR assays demonstrated that *HOXD11* reduced significantly in OA as compared with control (*P* < 0.05) (Fig. 5a). mRNA levels of the other *5′-HOXD* genes were not significantly different. To test the hypothesis that *HOXD11* expression modulates cartilage degradation in OA, we also measured *HOXD11* protein levels (Fig. 5b). WB showed a reduction in OA as compared with control (*P* < 0.05).

### *HOXD11* overexpression reversed effect of human OA chondrocytes

**Fig. 6:**
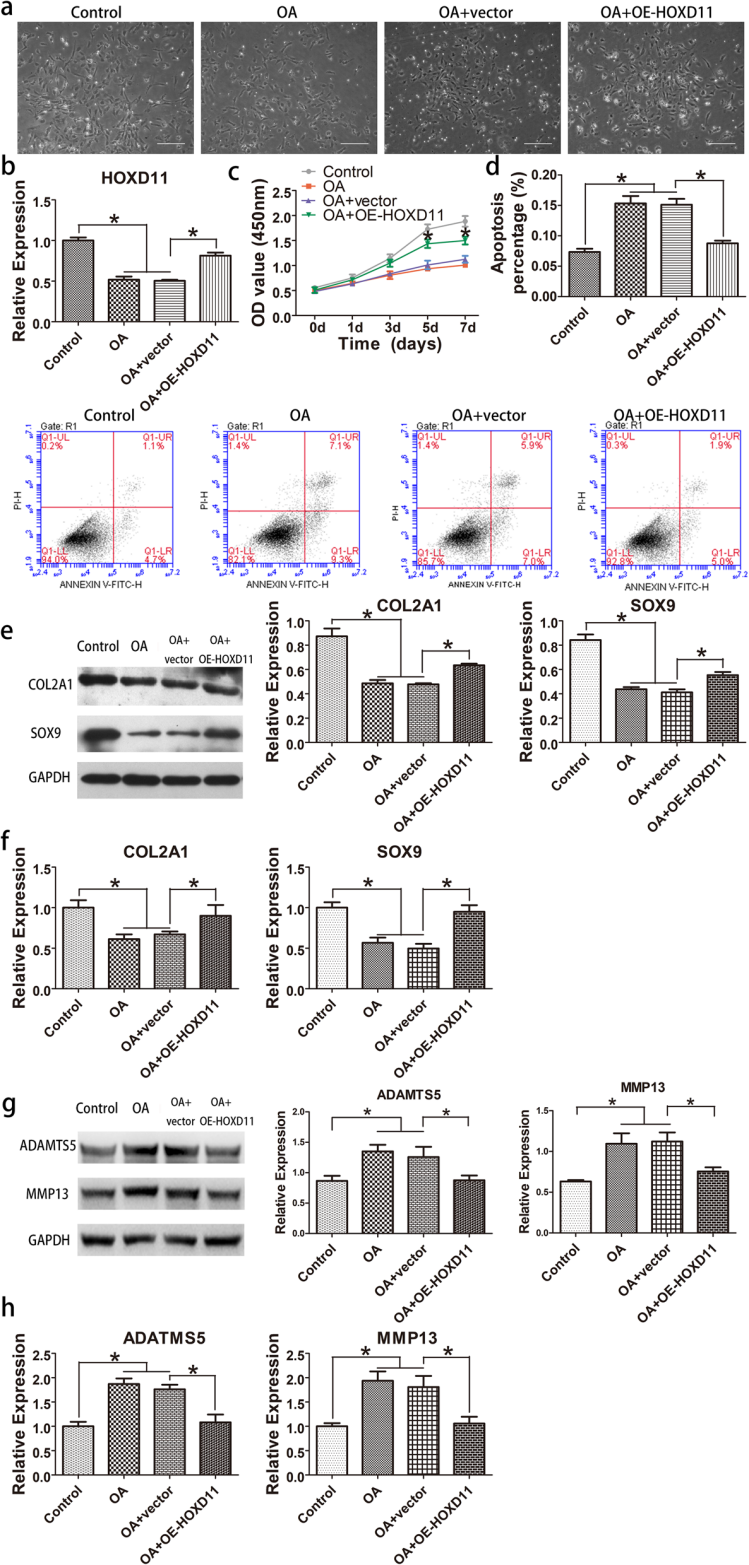
Overexpression of *HOXD11* in OA cells reverses the effects. **a** The cultivated chondrocyte cells in 60 mm plates (magnification, × 100). Scale bars: 200 μM. **b** qRT-PCR was performed to evaluate *HOXD11* mRNA expression. **c** CCK-8 was conducted to evaluate proliferation. **d** Flow cytometry was performed to evaluate cell apoptosis. **e** WB was performed to evaluate expression of SOX9 and COL2A1. **f** qRT-PCR was conducted to evaluate SOX9 and COL2A1 mRNA levels. **g** WB was used to quantify MMP13 and ADAMTS5 expression. **h** qRT-PCR was utilized to quantify MMP13 and ADAMTS5 mRNA levels. *P < 0.05, **P < 0.01 versus control or OA + vector

To determine if *HOXD11* exerts its role via regulating SOX9 expression, *HOXD11* overexpression experiments were conducted. *HOXD11* overexpression was achieved by lentivirus transfection according to qRT-PCR analysis (Fig. 6b). Proliferation impact in OA chondrocytes was reversed by *HOXD11*, as determined by CCK-8 analysis (Fig. 6c). Moreover, *HOXD11* overexpression inhibited cell apoptosis in OA chondrocytes, as shown by flow cytometry (Fig. 6d). SOX9 and COL2A1 expressions were also detected. qRT-PCR and WB results suggested that *HOXD11* enhanced SOX9 and COL2A1 expressions (Fig. 6e, f), which were initially reduced. Furthermore, qRT-PCR and WB were conducted to detect ADAMTS5 and MMP13 expressions. The findings exhibited that *HOXD11* inhibited ADAMTS5 and MMP13 expression (Fig. 6g, h), which were initially upregulated.

### Formed aberrant subchondral bone and downregulated expression of SOX9 and *HOXD11* in OA cartilage

**Fig. 7:**
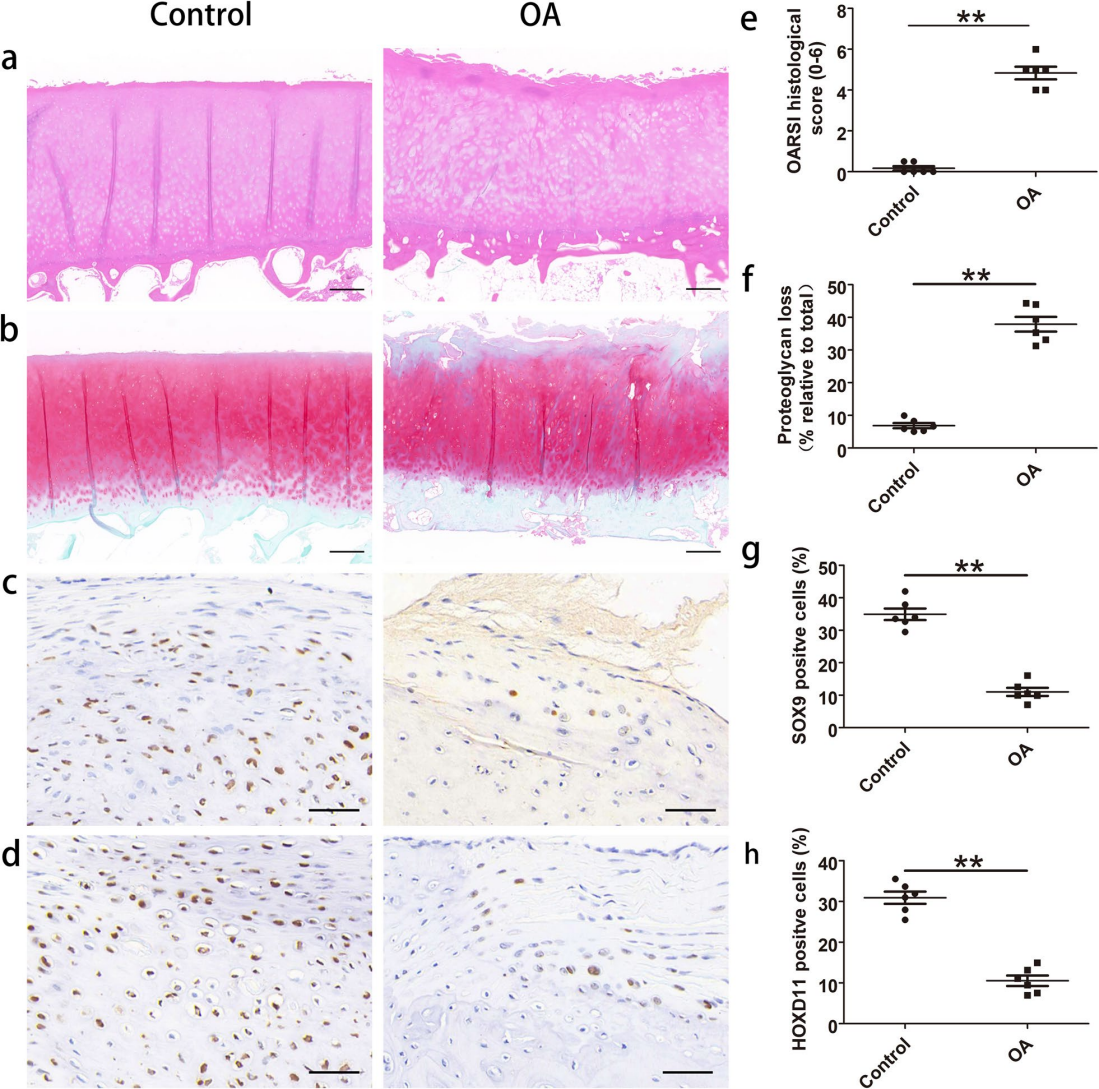
Cartilage staining and immunohistochemistry assay of SOX9 and *HOXD11* expression. **a** and **b** H&E and SOFG were used to stain the cartilage and assess the cartilage damage (original magnification × 40). Scale bars: 500 μM. **c** and **d** Immunohistochemistry of SOX9 and *HOXD11* in human normal and OA knee joint cartilage (original magnification × 400). Scale bars: 50 μM. **e** OARSI histological score of OA severity in normal and OA knee joint cartilage. **f** Quantification of proteoglycan loss in normal and OA knee joint cartilage. **g** and h SOX9, *HOXD11*-positive cells were quantified in normal and OA knee joint cartilage. *P < 0.05, **P < 0.01 versus control

**Fig. 8:**
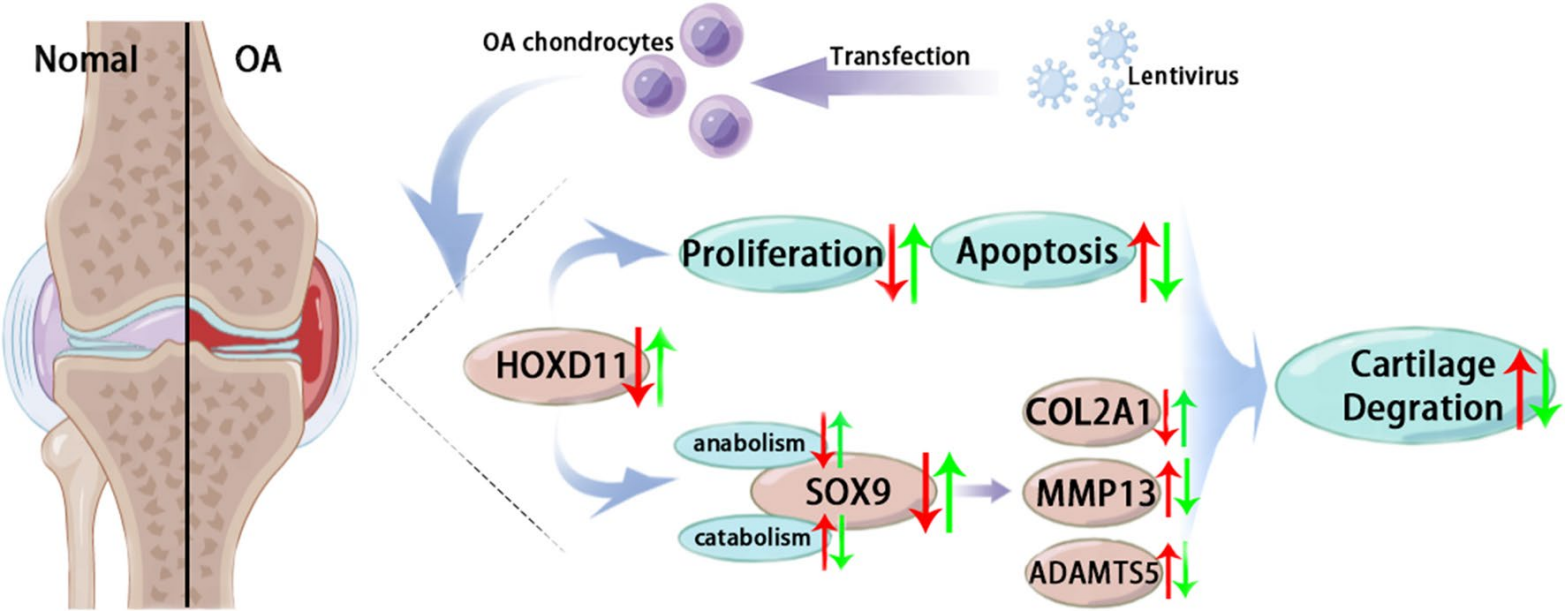
Model for inhibition of *HOXD11* promotes cartilage degradation and induces osteoarthritis development (red arrow). In OA chondrocytes, the decreased expression of *HOXD11* inhibits cell proliferation and enhances apoptosis. *HOXD11* downregulates SOX9 via inducing catabolism and restraining anabolism, leading to the upregulation of MMP13 and ADAMTS5, which downregulates COL2A1, ultimately promoting cartilage degradation and inducing osteoarthritis development. Lentivirus-mediated *HOXD11* overexpression reverses these effects (green arrow)

To measure aberrant subchondral bone formation in OA cartilage, we performed the experiments of H&E staining and SOFG staining (Fig. 7a, b). The results showed that the OARSI histological score was 0.1667 in controls and 4.833 in the OA group (Fig. 7e). For proteoglycan loss (% relative to total) experiments, the results showed that 6.843 percentage of proteoglycan loss in the controls, and 37.9 percentage in OA cartilage (Fig. 7f) (Fig. 8).

We further detected the expression of SOX9 and *HOXD11* in knee joint cartilage utilizing immunohistochemistry. Findings displayed that SOX9 expression was significantly reduced in OA cartilage as relative with controls (*P* < 0.01) (Fig. 7c, g). Similarly, *HOXD11* expression was reduced in OA cartilage (Fig. 7d, h).

## Discussion

During OA development, chondrocyte is characterized by elevated apoptosis, cytokine production, and matrix degeneration [26–30]. Chondrocyte apoptosis in OA cartilage has been recognized as one of the most crucial factors in pathophysiology of OA illness process [26, 31]. This study illustrated significantly inhibited cell proliferation while markedly increased cell apoptosis in human OA chondrocytes (Fig. 1), indicating the participation of cell apoptosis in OA development, consistent with previous studies [32, 33].

SOX9 is a master transcription regulator required for chondrogenesis, which controls condensation as well as differentiation of mesenchymal cells by cooperating with other cartilage-specific factors, such as COL2A1 [25], aggrecan [34], and cartilage link protein [35]. COL2A1, regulated by SOX9, serves as the master component in ECM; thus, it is recognized as a target gene of SOX9 in vivo [36]. This study found that SOX9 and COL2A1 expression was noticeably decreased in human OA chondrocytes, demonstrating that the signaling pathway of SOX9-COL2A1 might have an essential role in cartilage degradation and OA development [24].

*5′-HOXD* genes roles in many diseases have been reported, especially in cancer and congenital malformations, and they are therefore emerging as novel pharmacological targets [37–40]. There is increasing evidence that *HOXD9*, one of the *5′-HOXD* genes, has a role in development of normal joints in early stages and the pathological process of arthritis [22, 23, 41, 42]. Our previous study has found that *HOXD9* has a close relationship with chondrogenesis by regulating SOX9 and its down-target, COL2A1 [24]. In this study, four DEGs in the GSE169077 dataset were screened in OA and normal cartilage samples based on the differential analysis in the GEO database. Based on the analysis (Fig. 4 and Table 2), *HOXD11* was downregulated and identified as DE in OA. To verify whether *HOXD11* was downregulated in OA chondrocytes, we detected the mRNA levels of *5′-HOXD* genes utilizing qRT-PCR analysis. We found that *HOXD11* mRNA levels were reduced in OA, which supported the findings of bioinformatic analysis (Fig. 5). WB and immunohistochemistry results also showed that *HOXD11* protein levels decreased in OA chondrocytes, which corresponded with qRT-PCR results. Conclusively, *HOXD11* was markedly decreased in the human OA chondrocytes in vitro, consistent with the cartilage-specific factors of SOX9 and COL2A1. To further understand the relationship between *HOXD11* and SOX9-COL2A1, we performed the *HOXD11* overexpression experiments and found an increased expression of SOX9 and COL2A1, which reversed downregulating effects. These results strongly show that *HOXD11* may have a key involvement in cartilage degradation and the development of OA via the SOX9-COL2A1 signaling pathway. Interestingly, this inference differs from our previous conclusion about *HOXD9* and cartilage formation [24]. We attribute the reason to the difference in study species and cell type. Besides, this inference just showed the overlapping function of the *5′-HOXD* genes [21].

In the cartilage degradation of OA, MMPs and ADAMTSs are examples of matrix-degrading enzymes responsible for degrading ECM. Among the MMPs, MMP13 is primary collagenase in OA cartilage [43, 44]. Recently, research showed that ADAMTS5 is superior to ADAMTS4 at aggrecan breakdown of the experimental models and human OA cartilage [12]. We found the upregulated expression of MMPs (MMP3 and MMP13) and ADAMTSs (ADAMTS4 and ADAMTS5) in OA-cultured chondrocytes, similar to the reports before [31] (Fig. 3). In the *HOXD11* overexpression experiments, we found ADAMTS5 and MMP13 expression were reduced, which reversed upregulating effects. These findings strongly show that *HOXD11* may have a key involvement in cartilage degradation and *HOXD11* overexpression may play an anti-inflammatory and anabolic effect in cultivated chondrocytes.

Nonetheless, this study had a number of limitations. Firstly, *HOXD11* knockdown effect in OA chondrocytes was not identified, despite *HOXD11* overexpression results were displayed in our study, which necessitates further research. Second, the molecular mechanism is relatively simple to explore in the present study. Whether the *HOXD11* has a related signaling pathway and whether the pathway is related to osteoarthritis, it needs further research on the relationship between the *HOXD11* and the signaling pathway.

To conclude, this study demonstrated inhibited expression of *HOXD11* in human OA chondrocytes and cartilages, parallel with observed expression levels of SOX9 and COL2A1. Lentivirus-mediated overexpression of *HOXD11* reversed OA effects on chondrocyte proliferation and apoptosis and reduced cartilage damage. Our findings proposed that *HOXD11* has a critical role in OA development as well as *HOXD11* overexpression could be effective therapeutic strategy for preventing OA cartilage degradation.
